# Methyl 2-{[(3-methyl-5-oxo-1-phenyl-4,5-dihydro-1*H*-pyrazol-4-yl­idene)(4-nitro­phen­yl)meth­yl]amino}-3-phenyl­propano­ate

**DOI:** 10.1107/S1600536812010343

**Published:** 2012-03-14

**Authors:** Xin Zhang, Chen Sun, Fei-ran Li, Hua Zhang

**Affiliations:** a College of Chemistry, Tianjin Normal University, Tianjin 300387, People’s Republic of China

## Abstract

The mol­ecule of the title compound, C_27_H_24_N_4_O_5_, exists in the keto–enamine tautomeric form, stabilized by an intra­molecular N—H⋯O hydrogen bond. An intra­molecular C—H⋯·O hydrogen bond also occurs. In the crystal, C—H⋯O hydrogen bonds link the mol­ecules into chains.

## Related literature

For general background to Schiff bases in coordination chemistry, see: Wu *et al.* (1993 ▶); Harrop *et al.* (2003 ▶); Habibi *et al.* (2007 ▶). For anti­bacterial properties of Schiff bases derived from 4-acyl-5-pyrazolone and their metal complexes, see: Li *et al.* (1997 ▶, 2004 ▶). For the anti­bacterial and biological activity of amino acid esters, see: Xiong *et al.* (1993 ▶). For related structures, see: Wang *et al.* (2003 ▶); Zhang *et al.* (2005 ▶). For synthetic details, see: Remya *et al.* (2005 ▶). For standard bond lengths, see: Allen *et al.* (1987 ▶). 
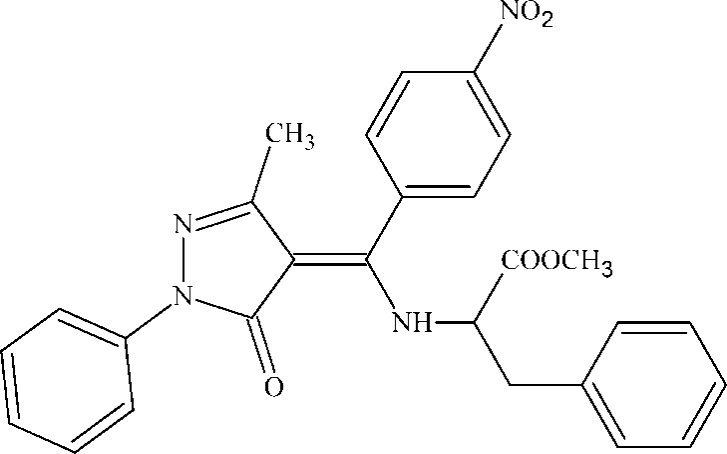


## Experimental

### 

#### Crystal data

C_27_H_24_N_4_O_5_
*M* *_r_* = 484.50
Monoclinic, 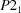

*a* = 6.7713 (16) Å
*b* = 8.917 (2) Å
*c* = 20.339 (5) Å
β = 92.489 (4)°
*V* = 1226.9 (5) Å^3^
*Z* = 2
Mo *K*α radiation
μ = 0.09 mm^−1^
*T* = 296 K
0.20 × 0.16 × 0.12 mm

#### Data collection

Bruker APEXII CCD diffractometer
Absorption correction: multi-scan (*SADABS*; Bruker, 2007 ▶) *T*_min_ = 0.980, *T*_max_ = 0.989
6305 measured reflections
2315 independent reflections
1855 reflections with *I* > 2σ(*I*)
*R*_int_ = 0.021

#### Refinement

*R*[*F* ^2^ > 2σ(*F* ^2^)] = 0.040
*wR*(*F* ^2^) = 0.106
*S* = 1.07
2315 reflections
327 parameters
1 restraint
H-atom parameters constrained
Δρ_max_ = 0.26 e Å^−3^
Δρ_min_ = −0.15 e Å^−3^

### 

Data collection: *APEX2* (Bruker, 2007 ▶); cell refinement: *SAINT* (Bruker, 2007 ▶); data reduction: *SAINT*; program(s) used to solve structure: *SHELXS97* (Sheldrick, 2008 ▶); program(s) used to refine structure: *SHELXL97* (Sheldrick, 2008 ▶); molecular graphics: *SHELXTL* (Sheldrick, 2008 ▶); software used to prepare material for publication: *SHELXTL*.

## Supplementary Material

Crystal structure: contains datablock(s) I, global. DOI: 10.1107/S1600536812010343/yk2041sup1.cif

Structure factors: contains datablock(s) I. DOI: 10.1107/S1600536812010343/yk2041Isup2.hkl

Supplementary material file. DOI: 10.1107/S1600536812010343/yk2041Isup3.cml

Additional supplementary materials:  crystallographic information; 3D view; checkCIF report

## Figures and Tables

**Table 1:** Hydrogen-bond geometry (Å, °)

*D*—H⋯*A*	*D*—H	H⋯*A*	*D*⋯*A*	*D*—H⋯*A*
N4—H4⋯O1	0.86	2.04	2.738 (4)	138
C1—H1⋯O1	0.93	2.41	3.001 (4)	121
C20—H20*A*⋯O1^i^	0.96	2.55	3.385 (5)	145

Symmetry code: (i) 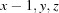.

**Fig. 1.**
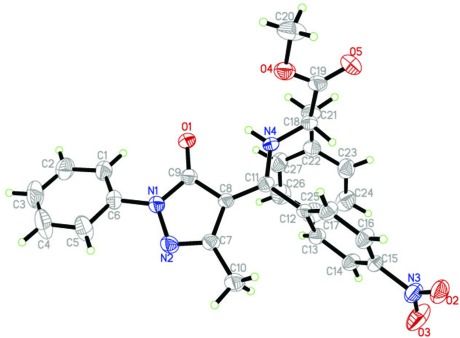
The molecular structure of the title molecule with the atom-numbering scheme. Displacement ellipsoids are drawn at the 30% probability level.

**Fig. 2.**
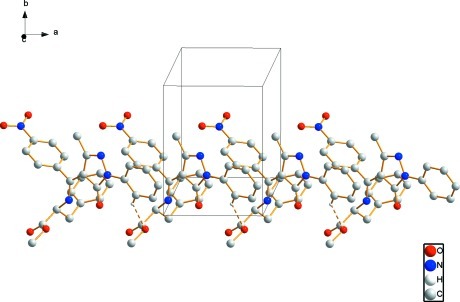
The chain formed by the intermolecular C—H···O hydrogen bonds (shown by dashed lines).

## supplementary crystallographic information

### Comment

In recent years, Schiff bases play an important role in the development of coordination chemistry related to catalysis and enzymatic reactions, magnetism, and molecular architectures (Wu *et al.*, 1993; Harrop *et al.*, 2003; Habibi *et al.*, 2007). In recent years, the Schiff bases derived from 4–acyl–5–pyrazolone and their metal complexes have been studied widely for their high antibacterial activity (Li *et al.*, 1997, 2004). Both 1–phenyl–3–methyl–4–(*p*–nitro–benzyl)–5–pyrazolone and its metal complexes are widely used and well known for their analgetic activity (Remya *et al.*, 2005). Amino acid esters also demonstrate high antibacterial and biological activity (Xiong *et al.*, 1993). Structure of Schiff base derived from 4–acyl–5–pyrazolone and amino acid ester, closely related to the title compound, has been reported (Zhang *et al.*, 2005).

The molecular structure of the title compound is presented in Fig. 1, and the numerical results are given in tables below. Atoms O1, C9, C8, C11 and N4 form a plane, the largest deviation being 0.021 (4) Å for atom C11. The dihedral angle between this mean plane and the pyrazolone ring is 1.2 (1)°, indicating that they are essentially coplanar, as seen in 4–{[3,4–dihydro–5–methyl–3–oxo–2–phenyl–2H–pyrazol–4–ylidene]–(phenyl)methyl]amino}–1,5–dimethyl–2–phenyl–1H–pyrazol–3(2*H*)–one [3.56 (3)°; Wang *et al.*, 2003]. The bond lengths within central part of the molecule lie between typical single- and double- bond lengths, indicating extensive conjugation. A strong intramolecular N—H···O hydrogen bond is observed, stabilizing the enamine-keto tautomeric form. In the crystal structure, intermolecular C1—H1···O4 hydrogen bonds link the molecules into chains, shown in Fig. 2.

### Experimental

The title compound was synthesized by refluxing a mixture of 1–phenyl–3—methyl–4–(p–nitro–benzyl)–5–pyrazolone (15 mmol) (Remya *et al.*, 2005) and phenylalanine methyl ester (15 mmol) in ethanol (100 ml) for about 5 h. The product was recrystallized from ethanol, affording pale yellow crystals suitable for *X*–ray analysis.

### Refinement

All H atoms were positioned geometrically with N—H = 0.86 Å and C—H = 0.93–0.98 Å, and constrained to ride on their parent atoms, with *U*_iso_(H) = *xU*_eq_(C,N), where *x* = 1.5 for methyl H and *x* = 1.2 for other H atoms.

### Crystal data

**Table:**

C_27_H_24_N_4_O_5_	*F*(000) = 508
*M**_r_* = 484.50	*D*_x_ = 1.312 Mg m^−^^3^
Monoclinic, *P*2_1_	Mo *K*α radiation, λ = 0.71073 Å
Hall symbol: P 2yb	Cell parameters from 2125 reflections
*a* = 6.7713 (16) Å	θ = 2.5–22.4°
*b* = 8.917 (2) Å	µ = 0.09 mm^−^^1^
*c* = 20.339 (5) Å	*T* = 296 K
β = 92.489 (4)°	Block, colourless
*V* = 1226.9 (5) Å^3^	0.20 × 0.16 × 0.12 mm
*Z* = 2	

### Data collection

**Table:**

Bruker APEXII CCD diffractometer	2315 independent reflections
Radiation source: fine-focus sealed tube	1855 reflections with *I* > 2σ(*I*)
Graphite monochromator	*R*_int_ = 0.021
φ and ω scan	θ_max_ = 25.0°, θ_min_ = 2.0°
Absorption correction: multi-scan (*SADABS*; Bruker, 2007)	*h* = −8→7
*T*_min_ = 0.980, *T*_max_ = 0.989	*k* = −10→10
6305 measured reflections	*l* = −24→16

### Refinement

**Table:**

Refinement on *F*^2^	Primary atom site location: structure-invariant direct methods
Least-squares matrix: full	Secondary atom site location: difference Fourier map
*R*[*F*^2^ > 2σ(*F*^2^)] = 0.040	Hydrogen site location: inferred from neighbouring sites
*wR*(*F*^2^) = 0.106	H-atom parameters constrained
*S* = 1.07	*w* = 1/[σ^2^(*F*_o_^2^) + (0.0582*P*)^2^ + 0.0914*P*] where *P* = (*F*_o_^2^ + 2*F*_c_^2^)/3
2315 reflections	(Δ/σ)_max_ < 0.001
327 parameters	Δρ_max_ = 0.26 e Å^−^^3^
1 restraint	Δρ_min_ = −0.15 e Å^−^^3^

### Special details

**Table:**

Geometry. All e.s.d.'s (except the e.s.d. in the dihedral angle between two l.s. planes) are estimated using the full covariance matrix. The cell e.s.d.'s are taken into account individually in the estimation of e.s.d.'s in distances, angles and torsion angles; correlations between e.s.d.'s in cell parameters are only used when they are defined by crystal symmetry. An approximate (isotropic) treatment of cell e.s.d.'s is used for estimating e.s.d.'s involving l.s. planes.
Refinement. Refinement of *F*^2^ against ALL reflections. The weighted *R*-factor *wR* and goodness of fit *S* are based on *F*^2^, conventional *R*-factors *R* are based on *F*, with *F* set to zero for negative *F*^2^. The threshold expression of *F*^2^ > σ(*F*^2^) is used only for calculating *R*-factors(gt) *etc*. and is not relevant to the choice of reflections for refinement. *R*-factors based on *F*^2^ are statistically about twice as large as those based on *F*, and *R*- factors based on ALL data will be even larger.

### Fractional atomic coordinates and isotropic or equivalent isotropic displacement parameters (Å^2^)

**Table:**

	*x*	*y*	*z*	*U*_iso_*/*U*_eq_	
O1	0.2384 (4)	−0.1136 (2)	0.34497 (13)	0.0631 (7)	
O2	−0.7694 (4)	0.4350 (4)	0.12919 (15)	0.0803 (9)	
O3	−0.5325 (5)	0.4977 (5)	0.06804 (18)	0.1058 (12)	
O4	−0.3079 (4)	−0.3075 (3)	0.31388 (15)	0.0683 (7)	
O5	−0.4842 (5)	−0.3580 (4)	0.22187 (18)	0.1000 (11)	
N1	0.3237 (4)	0.1290 (3)	0.38007 (13)	0.0506 (7)	
N2	0.2558 (4)	0.2758 (3)	0.36907 (15)	0.0579 (7)	
N3	−0.5986 (4)	0.4262 (4)	0.11306 (16)	0.0645 (8)	
N4	−0.0863 (4)	−0.0999 (3)	0.26012 (15)	0.0551 (7)	
H4	−0.0086	−0.1510	0.2861	0.066*	
C1	0.6183 (5)	−0.0145 (5)	0.4130 (2)	0.0709 (11)	
H1	0.5785	−0.0909	0.3842	0.085*	
C2	0.7941 (6)	−0.0259 (6)	0.4492 (2)	0.0879 (14)	
H2	0.8727	−0.1104	0.4444	0.105*	
C3	0.8550 (7)	0.0833 (8)	0.4916 (3)	0.1011 (17)	
H3	0.9744	0.0747	0.5156	0.121*	
C4	0.7377 (8)	0.2064 (9)	0.4983 (3)	0.118 (2)	
H4A	0.7776	0.2803	0.5283	0.142*	
C5	0.5612 (7)	0.2250 (6)	0.4621 (2)	0.0901 (15)	
H5	0.4852	0.3112	0.4661	0.108*	
C6	0.5025 (5)	0.1097 (4)	0.41960 (16)	0.0534 (8)	
C7	0.1051 (5)	0.2653 (4)	0.32700 (17)	0.0508 (8)	
C8	0.0671 (4)	0.1120 (3)	0.30814 (15)	0.0437 (7)	
C9	0.2132 (4)	0.0246 (4)	0.34512 (16)	0.0474 (8)	
C10	0.0044 (6)	0.4069 (4)	0.3050 (3)	0.0824 (13)	
H10A	0.0556	0.4893	0.3310	0.124*	
H10B	−0.1352	0.3979	0.3106	0.124*	
H10C	0.0281	0.4245	0.2595	0.124*	
C11	−0.0733 (4)	0.0480 (4)	0.26523 (15)	0.0449 (8)	
C12	−0.2120 (4)	0.1434 (3)	0.22373 (15)	0.0423 (7)	
C13	−0.1507 (5)	0.2096 (4)	0.16670 (17)	0.0514 (8)	
H13	−0.0236	0.1915	0.1530	0.062*	
C14	−0.2753 (5)	0.3019 (4)	0.12978 (17)	0.0545 (9)	
H14	−0.2339	0.3465	0.0914	0.065*	
C15	−0.4630 (4)	0.3264 (4)	0.15121 (17)	0.0489 (8)	
C16	−0.5284 (5)	0.2614 (4)	0.20677 (18)	0.0593 (9)	
H16	−0.6562	0.2791	0.2199	0.071*	
C17	−0.4023 (5)	0.1686 (4)	0.24343 (17)	0.0564 (9)	
H17	−0.4454	0.1231	0.2814	0.068*	
C18	−0.2165 (5)	−0.1856 (4)	0.21573 (19)	0.0591 (9)	
H18	−0.3020	−0.1133	0.1920	0.071*	
C19	−0.3515 (6)	−0.2928 (4)	0.2507 (2)	0.0636 (10)	
C20	−0.4402 (7)	−0.4046 (5)	0.3506 (3)	0.0948 (15)	
H20A	−0.5692	−0.3599	0.3509	0.142*	
H20B	−0.3884	−0.4159	0.3950	0.142*	
H20C	−0.4494	−0.5012	0.3299	0.142*	
C21	−0.0987 (6)	−0.2697 (4)	0.1635 (2)	0.0731 (11)	
H21A	−0.0013	−0.3339	0.1857	0.088*	
H21B	−0.1884	−0.3331	0.1375	0.088*	
C22	0.0040 (6)	−0.1652 (4)	0.1186 (2)	0.0635 (10)	
C23	−0.0986 (7)	−0.0965 (5)	0.0657 (2)	0.0777 (12)	
H23	−0.2311	−0.1194	0.0570	0.093*	
C24	−0.0046 (8)	0.0060 (6)	0.0261 (2)	0.0874 (14)	
H24	−0.0743	0.0508	−0.0091	0.105*	
C25	0.1903 (8)	0.0413 (6)	0.0387 (3)	0.0911 (14)	
H25	0.2528	0.1109	0.0126	0.109*	
C26	0.2911 (7)	−0.0259 (6)	0.0894 (3)	0.0903 (14)	
H26	0.4238	−0.0028	0.0976	0.108*	
C27	0.2016 (6)	−0.1274 (5)	0.1291 (2)	0.0816 (13)	
H27	0.2746	−0.1717	0.1637	0.098*	

### Atomic displacement parameters (Å^2^)

**Table:**

	*U*^11^	*U*^22^	*U*^33^	*U*^12^	*U*^13^	*U*^23^
O1	0.0668 (15)	0.0396 (15)	0.0809 (17)	0.0042 (11)	−0.0176 (12)	0.0071 (12)
O2	0.0508 (15)	0.087 (2)	0.103 (2)	0.0157 (14)	−0.0078 (14)	0.0168 (17)
O3	0.086 (2)	0.128 (3)	0.104 (2)	0.033 (2)	0.0136 (17)	0.062 (2)
O4	0.0676 (15)	0.0495 (15)	0.088 (2)	−0.0010 (13)	0.0039 (14)	0.0021 (14)
O5	0.093 (2)	0.072 (2)	0.132 (3)	−0.0315 (19)	−0.0316 (19)	0.004 (2)
N1	0.0474 (14)	0.0488 (16)	0.0552 (16)	−0.0002 (13)	−0.0033 (12)	−0.0010 (14)
N2	0.0578 (16)	0.0465 (16)	0.0688 (19)	0.0048 (14)	−0.0034 (14)	−0.0125 (14)
N3	0.0571 (18)	0.064 (2)	0.072 (2)	0.0105 (15)	−0.0038 (15)	0.0147 (17)
N4	0.0621 (17)	0.0311 (15)	0.0703 (19)	0.0030 (13)	−0.0170 (14)	0.0037 (13)
C1	0.066 (2)	0.071 (3)	0.074 (3)	0.007 (2)	−0.0150 (19)	−0.001 (2)
C2	0.064 (2)	0.095 (4)	0.102 (3)	0.009 (3)	−0.020 (2)	0.012 (3)
C3	0.062 (3)	0.145 (5)	0.094 (4)	−0.008 (3)	−0.028 (2)	0.003 (4)
C4	0.089 (4)	0.153 (6)	0.109 (4)	−0.005 (4)	−0.039 (3)	−0.045 (4)
C5	0.073 (3)	0.104 (4)	0.092 (3)	0.004 (2)	−0.018 (2)	−0.034 (3)
C6	0.0458 (17)	0.070 (2)	0.0444 (19)	−0.0047 (18)	0.0009 (13)	0.0002 (17)
C7	0.0468 (18)	0.0410 (17)	0.065 (2)	0.0064 (15)	0.0035 (15)	−0.0057 (16)
C8	0.0424 (16)	0.0355 (17)	0.0530 (19)	0.0032 (14)	−0.0005 (13)	0.0001 (14)
C9	0.0440 (18)	0.046 (2)	0.052 (2)	0.0001 (14)	0.0027 (14)	−0.0001 (15)
C10	0.080 (3)	0.046 (2)	0.119 (4)	0.016 (2)	−0.025 (2)	−0.018 (2)
C11	0.0440 (17)	0.0440 (19)	0.0469 (19)	0.0036 (14)	0.0046 (14)	0.0020 (14)
C12	0.0439 (16)	0.0315 (15)	0.0514 (18)	0.0007 (13)	0.0008 (13)	−0.0007 (14)
C13	0.0421 (17)	0.0472 (18)	0.065 (2)	0.0044 (15)	0.0073 (15)	0.0034 (17)
C14	0.0540 (19)	0.053 (2)	0.057 (2)	0.0031 (16)	0.0082 (15)	0.0122 (16)
C15	0.0441 (16)	0.0418 (18)	0.060 (2)	0.0067 (14)	−0.0031 (15)	0.0012 (15)
C16	0.0439 (17)	0.069 (2)	0.065 (2)	0.0101 (18)	0.0069 (15)	0.011 (2)
C17	0.0525 (18)	0.060 (2)	0.057 (2)	0.0054 (17)	0.0089 (15)	0.0169 (16)
C18	0.067 (2)	0.0341 (17)	0.075 (3)	0.0029 (16)	−0.0178 (19)	−0.0030 (17)
C19	0.064 (2)	0.0346 (17)	0.092 (3)	0.0031 (17)	−0.007 (2)	−0.0013 (19)
C20	0.088 (3)	0.063 (3)	0.136 (4)	−0.007 (2)	0.036 (3)	0.011 (3)
C21	0.086 (3)	0.045 (2)	0.087 (3)	0.0055 (19)	−0.007 (2)	−0.009 (2)
C22	0.072 (2)	0.047 (2)	0.070 (2)	0.0085 (18)	−0.0045 (19)	−0.0138 (18)
C23	0.086 (3)	0.076 (3)	0.070 (3)	0.012 (2)	−0.016 (2)	−0.016 (2)
C24	0.117 (4)	0.091 (4)	0.053 (2)	0.014 (3)	−0.003 (2)	−0.005 (2)
C25	0.106 (4)	0.088 (3)	0.081 (3)	0.011 (3)	0.025 (3)	−0.003 (3)
C26	0.067 (3)	0.091 (3)	0.113 (4)	0.007 (3)	0.012 (3)	−0.003 (3)
C27	0.069 (3)	0.079 (3)	0.096 (3)	0.017 (2)	−0.011 (2)	0.002 (3)

### Geometric parameters (Å, º)

**Table:**

O1—C9	1.244 (4)	C11—C12	1.500 (4)
O2—N3	1.218 (4)	C12—C13	1.381 (4)
O3—N3	1.217 (4)	C12—C17	1.384 (4)
O4—C19	1.313 (5)	C13—C14	1.378 (5)
O4—C20	1.473 (5)	C13—H13	0.9300
O5—C19	1.201 (4)	C14—C15	1.379 (4)
N1—C9	1.374 (4)	C14—H14	0.9300
N1—N2	1.403 (4)	C15—C16	1.361 (5)
N1—C6	1.434 (4)	C16—C17	1.384 (5)
N2—C7	1.306 (4)	C16—H16	0.9300
N3—C15	1.474 (4)	C17—H17	0.9300
N4—C11	1.326 (4)	C18—C19	1.520 (6)
N4—C18	1.452 (4)	C18—C21	1.550 (6)
N4—H4	0.8600	C18—H18	0.9800
C1—C6	1.367 (6)	C20—H20A	0.9600
C1—C2	1.376 (5)	C20—H20B	0.9600
C1—H1	0.9300	C20—H20C	0.9600
C2—C3	1.352 (8)	C21—C22	1.497 (6)
C2—H2	0.9300	C21—H21A	0.9700
C3—C4	1.365 (8)	C21—H21B	0.9700
C3—H3	0.9300	C22—C27	1.387 (6)
C4—C5	1.386 (7)	C22—C23	1.396 (6)
C4—H4A	0.9300	C23—C24	1.391 (7)
C5—C6	1.391 (6)	C23—H23	0.9300
C5—H5	0.9300	C24—C25	1.370 (7)
C7—C8	1.440 (5)	C24—H24	0.9300
C7—C10	1.494 (5)	C25—C26	1.352 (7)
C8—C11	1.385 (4)	C25—H25	0.9300
C8—C9	1.445 (4)	C26—C27	1.371 (7)
C10—H10A	0.9600	C26—H26	0.9300
C10—H10B	0.9600	C27—H27	0.9300
C10—H10C	0.9600		
			
C19—O4—C20	116.1 (3)	C12—C13—H13	119.5
C9—N1—N2	112.5 (2)	C13—C14—C15	118.3 (3)
C9—N1—C6	129.5 (3)	C13—C14—H14	120.9
N2—N1—C6	117.7 (3)	C15—C14—H14	120.9
C7—N2—N1	106.2 (3)	C16—C15—C14	122.2 (3)
O3—N3—O2	123.6 (3)	C16—C15—N3	118.5 (3)
O3—N3—C15	118.1 (3)	C14—C15—N3	119.4 (3)
O2—N3—C15	118.2 (3)	C15—C16—C17	119.1 (3)
C11—N4—C18	127.5 (3)	C15—C16—H16	120.4
C11—N4—H4	116.3	C17—C16—H16	120.4
C18—N4—H4	116.3	C16—C17—C12	120.1 (3)
C6—C1—C2	119.6 (4)	C16—C17—H17	119.9
C6—C1—H1	120.2	C12—C17—H17	119.9
C2—C1—H1	120.2	N4—C18—C19	113.7 (3)
C3—C2—C1	121.3 (5)	N4—C18—C21	111.3 (3)
C3—C2—H2	119.3	C19—C18—C21	110.8 (3)
C1—C2—H2	119.3	N4—C18—H18	106.9
C2—C3—C4	118.7 (4)	C19—C18—H18	106.9
C2—C3—H3	120.6	C21—C18—H18	106.9
C4—C3—H3	120.6	O5—C19—O4	124.1 (4)
C3—C4—C5	122.3 (5)	O5—C19—C18	121.9 (4)
C3—C4—H4A	118.8	O4—C19—C18	114.0 (3)
C5—C4—H4A	118.8	O4—C20—H20A	109.5
C4—C5—C6	117.3 (5)	O4—C20—H20B	109.5
C4—C5—H5	121.4	H20A—C20—H20B	109.5
C6—C5—H5	121.4	O4—C20—H20C	109.5
C1—C6—C5	120.7 (3)	H20A—C20—H20C	109.5
C1—C6—N1	121.1 (3)	H20B—C20—H20C	109.5
C5—C6—N1	118.1 (4)	C22—C21—C18	112.6 (3)
N2—C7—C8	111.6 (3)	C22—C21—H21A	109.1
N2—C7—C10	117.9 (3)	C18—C21—H21A	109.1
C8—C7—C10	130.4 (3)	C22—C21—H21B	109.1
C11—C8—C7	132.0 (3)	C18—C21—H21B	109.1
C11—C8—C9	122.8 (3)	H21A—C21—H21B	107.8
C7—C8—C9	105.3 (3)	C27—C22—C23	117.2 (4)
O1—C9—N1	126.9 (3)	C27—C22—C21	121.8 (4)
O1—C9—C8	128.6 (3)	C23—C22—C21	120.9 (4)
N1—C9—C8	104.4 (3)	C24—C23—C22	120.6 (4)
C7—C10—H10A	109.5	C24—C23—H23	119.7
C7—C10—H10B	109.5	C22—C23—H23	119.7
H10A—C10—H10B	109.5	C25—C24—C23	120.3 (5)
C7—C10—H10C	109.5	C25—C24—H24	119.9
H10A—C10—H10C	109.5	C23—C24—H24	119.9
H10B—C10—H10C	109.5	C26—C25—C24	119.4 (5)
N4—C11—C8	120.0 (3)	C26—C25—H25	120.3
N4—C11—C12	118.9 (3)	C24—C25—H25	120.3
C8—C11—C12	121.1 (3)	C25—C26—C27	121.4 (5)
C13—C12—C17	119.4 (3)	C25—C26—H26	119.3
C13—C12—C11	120.7 (3)	C27—C26—H26	119.3
C17—C12—C11	119.8 (3)	C26—C27—C22	121.2 (4)
C14—C13—C12	120.9 (3)	C26—C27—H27	119.4
C14—C13—H13	119.5	C22—C27—H27	119.4
			
C9—N1—N2—C7	−0.7 (4)	N4—C11—C12—C17	81.3 (4)
C6—N1—N2—C7	173.7 (3)	C8—C11—C12—C17	−99.0 (4)
C6—C1—C2—C3	0.3 (7)	C17—C12—C13—C14	1.0 (5)
C1—C2—C3—C4	0.3 (9)	C11—C12—C13—C14	−177.5 (3)
C2—C3—C4—C5	−1.7 (10)	C12—C13—C14—C15	−0.1 (5)
C3—C4—C5—C6	2.4 (9)	C13—C14—C15—C16	−0.7 (5)
C2—C1—C6—C5	0.5 (6)	C13—C14—C15—N3	179.3 (3)
C2—C1—C6—N1	176.6 (4)	O3—N3—C15—C16	171.5 (4)
C4—C5—C6—C1	−1.7 (7)	O2—N3—C15—C16	−7.0 (5)
C4—C5—C6—N1	−178.0 (4)	O3—N3—C15—C14	−8.5 (5)
C9—N1—C6—C1	18.5 (5)	O2—N3—C15—C14	173.0 (3)
N2—N1—C6—C1	−154.8 (3)	C14—C15—C16—C17	0.6 (6)
C9—N1—C6—C5	−165.3 (4)	N3—C15—C16—C17	−179.4 (3)
N2—N1—C6—C5	21.4 (5)	C15—C16—C17—C12	0.3 (6)
N1—N2—C7—C8	−0.2 (4)	C13—C12—C17—C16	−1.1 (5)
N1—N2—C7—C10	−178.9 (3)	C11—C12—C17—C16	177.4 (3)
N2—C7—C8—C11	−179.3 (3)	C11—N4—C18—C19	−121.9 (4)
C10—C7—C8—C11	−0.9 (7)	C11—N4—C18—C21	112.2 (4)
N2—C7—C8—C9	1.0 (4)	C20—O4—C19—O5	−3.5 (5)
C10—C7—C8—C9	179.4 (4)	C20—O4—C19—C18	177.1 (3)
N2—N1—C9—O1	179.9 (3)	N4—C18—C19—O5	170.7 (4)
C6—N1—C9—O1	6.3 (5)	C21—C18—C19—O5	−63.1 (5)
N2—N1—C9—C8	1.3 (3)	N4—C18—C19—O4	−10.0 (4)
C6—N1—C9—C8	−172.3 (3)	C21—C18—C19—O4	116.3 (4)
C11—C8—C9—O1	0.4 (5)	N4—C18—C21—C22	−64.3 (4)
C7—C8—C9—O1	−179.9 (3)	C19—C18—C21—C22	168.2 (3)
C11—C8—C9—N1	179.0 (3)	C18—C21—C22—C27	97.0 (5)
C7—C8—C9—N1	−1.3 (3)	C18—C21—C22—C23	−80.1 (5)
C18—N4—C11—C8	−176.7 (3)	C27—C22—C23—C24	−0.4 (6)
C18—N4—C11—C12	3.1 (5)	C21—C22—C23—C24	176.9 (4)
C7—C8—C11—N4	−174.9 (4)	C22—C23—C24—C25	−0.3 (7)
C9—C8—C11—N4	4.7 (5)	C23—C24—C25—C26	0.9 (7)
C7—C8—C11—C12	5.3 (5)	C24—C25—C26—C27	−0.8 (7)
C9—C8—C11—C12	−175.1 (3)	C25—C26—C27—C22	0.1 (7)
N4—C11—C12—C13	−100.2 (4)	C23—C22—C27—C26	0.5 (6)
C8—C11—C12—C13	79.5 (4)	C21—C22—C27—C26	−176.8 (4)

### Hydrogen-bond geometry (Å, º)

**Table:**

*D*—H···*A*	*D*—H	H···*A*	*D*···*A*	*D*—H···*A*
N4—H4···O1	0.86	2.04	2.738 (4)	138
C1—H1···O1	0.93	2.41	3.001 (4)	121
C20—H20*A*···O1^i^	0.96	2.55	3.385 (5)	145

Symmetry code: (i) *x*−1, *y*, *z*.
